# Supplementary material for: Spatiotemporal differentiation and analysis of factors influencing high-quality development of resource-based cities: An empirical study based in China
Source: PLoS One. 2024 May 31;19(5):e0304460. doi: 10.1371/journal.pone.0304460 (PMC11142578; doi:10.1371/journal.pone.0304460)
Supplement: Attachment — Submitted filename: Response to Reviewers.docx [file pone.0304460.s002.docx]

Dear Editor,

We are grateful to you and the other reviewers for your critical comments and thoughtful suggestions on our manuscript. We have carefully revised the manuscript based on your feedbacks. The revised portions of the manuscript are shown in red. We hope the revised manuscript satisfies the standards of the journal.

Below, we provide point-by-point responses to reviewer#1and reviewer#2’s comments and questions. We hope that these revisions are satisfactory and the revised manuscript could meet the publishing standards of the *PLOS one*.

Thank you very much for your assistance with our paper.

**Responses to Reviewer #1:**

*1.Why the data was chosen just before 2018.*

**Response:** According to the reviewer's comments, an explanation was provided for the selection of data prior to 2018. The reason is as follows: due to the outbreak of COVID-19 in China in 2019, the data for 2019-2020 is relatively distorted, and some data for 2021-2022 has not yet been released, resulting in the final selection of data prior to 2018.

*2.The definition of a resource city started in 2007, so why don't the data start in 2007.*

**Response:** According to the reviewer's comments, an explanation was provided for the selection of data prior to 2018. The reasons are as follows: First, although the definition of resource-based cities began in 2007, China's attention to resource-based cities dates back earlier than 2007. In 2001, Fuxin was designated as the first resource-depleted city in China's national economic transformation pilot cities. Since then, China has begun to pay attention to the transformation and development of resource-based cities. Secondly, the reason why 2001 was not chosen as the starting year is that China's socialist market economy system was basically established after 2002, and the domestic institutional environment in China was relatively stable. In addition, at the end of 2001, China joined the World Trade Organization, so the external environment faced by China during the sample period was basically the same. Therefore, selecting 2003 as the starting year of the study is appropriate.

*3.five development concepts, refer to ?Please define such Chinese expressions.*

**Response:** According to the reviewer's comments, a thorough review was conducted on the connotations of the Five Development Concepts. The revisions are as follows:

The Five Development Concepts refer to the five development strategies proposed by the Communist Party of China in the context of the new era, namely innovation, coordination, green development, openness, and sharing. These concepts aim to guide the development of China's economy, politics, culture, and other fields, and promote China's transition to high-quality development [38]. Specifically, innovation refers to promoting technological and industrial innovation to improve the quality and efficiency of economic development; coordination refers to promoting coordinated development between urban and rural areas, industries, regions, and sectors; green development refers to promoting ecological civilization construction, protecting the environment, and achieving coordinated development between economic development and environmental protection; openness refers to deepening opening-up, actively participating in global economic governance, and promoting economic globalization; sharing refers to promoting social fairness and justice, strengthening social security, and achieving more equitable and inclusive distribution of development outcomes. The Five Development Concepts are interconnected and mutually reinforcing, forming an integrated whole with inherent connections [39].

*4.Separate the introduction from the literature review.*

**Response:** According to the reviewer's comments, the introduction should be separated from the literature review. The revised version is as follows:

Currently, research in academia on high-quality development mainly focuses on its connotation, meaning, and evaluation [7-9], and a unified evaluation index system for high-quality development has not yet been formed, especially for resource-based regions. There is a lack of research on the evaluation index system for high-quality development in resource-based regions. In addition, scholars often use the national, provincial, and urban agglomeration levels as the research areas, analyzing the transformation and development of resource-based cities from the perspectives of labor quality [10], environmental pollution [11, 12], and education quality [13], but relatively little research has been done on the factors that affect high-quality development. Based on this, this paper takes 113 resource-based prefecture-level cities in China from 2003 to 2018 as samples, and uses entropy weight-TOPSIS method and spatial correlation analysis to construct a high-quality development evaluation system, and studies the spatio-temporal evolution trend of high-quality development in different types of cities. At the same time, a spatial econometric model is used to explore the role of industrial structure, technological innovation, human capital, urbanization, environmental regulation, openness to the outside world, and infrastructure in high-quality development. Finally, high-quality development suggestions are proposed based on empirical results.

The contributions of this study are: (1) Focusing on the "Five Major Development Concepts," a comprehensive and generalized evaluation index system for high-quality development of resource-based cities is constructed from multiple perspectives, which fills the gap in the current research on the evaluation index system for high-quality development of resource-based cities; (2) Including the vast majority of resource-based cities in China in the research sample makes the sample region more comprehensive and the study more practical; (3) Addressing the problem of a lack of research on the factors that influence high-quality development in resource-based cities and the inadequate selection of variables, this study selects the influencing factors as comprehensively, scientifically, and purposefully as possible, thereby analyzing the obstacles that resource-based cities face in the process of high-quality development and providing solutions.

The rest of this study is structured as follows. Part 2 introduces the relevant literature on high-quality development in resource-based cities. Part 3 describes the research methods, study areas, data sources, and variable selection. Part 4 presents the research structure and analysis, including spatio-temporal evolution analysis of high-quality development in resource-based cities and empirical testing of influencing factors. Part 5 is the conclusion and policy implications of the study.

**Literature Review**

The academic community's understanding of the connotation of high-quality development has undergone a transformation from focusing solely on the quality of economic growth to development quality. Initially, people's understanding of high-quality development was mostly focused on economic development, and they believed that when measuring regional development, using only per capita GDP was not comprehensive enough, and that people's lives and social welfare should also be included [14,15]. As scholars have conducted more in-depth research on the connotation of high-quality development, they have gradually incorporated aspects such as ecological environment, society, and human capital into the evaluation index system for measuring regional high-quality development [16-18]. At this point, the academic community has reached a consensus on the definition of the connotation of high-quality development, generally believing that high-quality development is efficient and high-quality development that can meet people's material, social, and psychological needs. Currently, with the proposal of the five development concepts of innovation, coordination, green, openness, and sharing, the connotation of high-quality development has been further deepened [19]. Compared with the previous connotation of high-quality development, the five development concepts have more systematic and comprehensive characteristics, more emphasis on people-oriented development, and are more in line with the trend of the times, providing more comprehensive theoretical support for evaluating regional development. Regarding the evaluation of high-quality development, most scholars use evaluation methods such as entropy weight method [20-23] and TOPISI method [24, 25] to construct evaluation index systems and calculate the level of high-quality development, and a few scholars use methods such as DEA model [26] and coupling coordination degree analysis [27-29] to evaluate the development of regions with high quality. In addition, some scholars have conducted comparative studies on the relationship and differences between high-quality development and sustainable development, believing that early high-quality development was more focused on economic quality compared to sustainable development [30]. However, with the proposal of the five development concepts, the content and scope of high-quality development have continued to expand [31], leading to the gradual formation of a unified whole in which high-quality development and sustainable development are mutually coordinated and complementary.

The research on the mechanism of high-quality development of resource-based cities mainly focuses on institutional construction, financial support, and industrial structure. For example, Al-Ubaydli [32] and Wiens [33] found that to avoid the resource curse, it is possible to promote the transformation and development of resource-based cities through high-quality institutional design. Song Liying et al. [34] proposed to reduce the fiscal autonomy and expand the scale of special funds by studying the role of fiscal autonomy in total factor productivity for resource-based cities. Jin Bei [35] believes that the government plays an important role in high-quality development, especially in the western region, which relies heavily on government support. At the same time, he suggests that technological innovation is the vitality source of high-quality development. Yang et al. [36] found that the single industrial structure, inadequate attention to ecological protection, and low regulatory efficiency of mineral resource-based cities increase the transformation cost and are not conducive to high-quality development. In addition, regarding the development path of resource-based cities, Yang et al. [5] pointed out that the transformation and development of resource-based cities cannot be achieved without long-term financial and policy support from the government. By improving the high pollution problems of resource-based enterprises or forcing high-pollution enterprises to exit the market, resource-based cities can achieve a smooth transformation. Li et al. [37] proposed that resource-based cities should construct ecological environmental protection and ecological compensation mechanisms that consider the full life cycle of mineral resource development and utilization to coordinate sustainable development and ecological protection of resources.

*5.Research gaps and research implications should be given thoroughly.*

**Response** According to the reviewer's comments, research gaps and inspirations should be added to the conclusion and recommendation section. The revised version is as follows:

Based on our results, we propose the following policy recommendations: (1) Strengthen the driving force of innovation and cultivate new momentum for development. Strengthening the research and development of green technology [4] should help transform and upgrade traditional resource-based industries, and move the industrial structure from low-end to high-end [64]. Improve the technology innovation system and mechanism in resource-based cities and implement dynamic management, optimize the regional industrial structure. A focus on human capital investment to meet the development needs of strategic emerging industries can help cultivate the independent innovation ability of enterprises and realize the industrialization of high-tech [52]. (2) Strengthen interregional coordination and exchanges to achieve mutual promotion and expected progress. Promoting the effective circulation of resources between urban and rural areas can create good human capital conditions for upgrading industrial structures. (3) Strengthen the coordinated control of pollutants and promote green development. Improving the energy consumption of secondary industries and environmental pollution should control the negative spillover effect between cities, and strictly control the transfer of pollution to neighboring resource-based cities. Furthermore, the comprehensive use of desulfurization, low-carbon, energy-saving, and environmental protection technologies [65] can strengthen the coordinated control of environmental pollutants and improve the compensation mechanism for environmental governance. Additionally, effectively utilizing the advantages of resource extraction and deep processing technologies, combined with emerging industries, encouraging increased flow to the resource recycling industry, and high-end equipment manufacturing industry can aid in building a green, diversified, and complementary industrial system. (4) Coordinate the external and internal openings to improve the openness level. Strengthening investment attraction can help attract powerful foreign enterprises. Additionally, targeting investment in resource-saving and environment-friendly enterprises can bring further energy-saving, emission-reduction technologies, and advanced experience. Free flow between countries can strengthen cooperation in the fields of energy, financial industry, technological innovation, and emerging industries. (5) Build a shared development system to promote social equity. The construction of "5G" and other new infrastructure, with the help of "Internet +" thinking can improve the public service system, and strengthen education, medical care, culture, etc., and establish a sound shared development system. Further, infrastructure capital investment should be rationally allocated, to improve investment efficiency. (6) Improve the human capital level of resource-based cities, with a focus on optimizing its structure [55]. China's resource-based cities should rely on higher education to cultivate high-quality human capital, promote their transformation towards resource-based industries and development towards innovative industries, and promote the effective accumulation and optimal allocation of talent resources.

This paper provides a comprehensive examination and analysis of the high-quality development of resource-based cities based on existing data and theory. However, there are still limitations. In the future, further exploration will be conducted in two aspects: first, the reasons for the differences in the impact mechanisms studied in this paper and those of other scholars should be analyzed, and it may be related to the existence of threshold effects. Therefore, future research should focus on whether there are threshold effects in the impact mechanism. In addition, facing various factors such as the proposal of China's "dual carbon" goal and the construction of the "14th Five-Year Plan", there is still a large space for promoting and improving the high-quality development of resource-based cities. The next step of the research should take these factors into consideration and solve current and future practical problems.

*6.The results of the appropriate discussion are studied by comparison.*

**Response** According to the reviewer's comments, corresponding result discussions have been added to the results section of the article.

Dear Editor,

We are grateful to you and the other reviewers for your critical comments and thoughtful suggestions on our manuscript. We have carefully revised the manuscript based on your feedbacks. The revised portions of the manuscript are shown in red. We hope the revised manuscript satisfies the standards of the journal.

Below, we provide point-by-point responses to reviewer#2’s comments and questions. We hope that these revisions are satisfactory and the revised manuscript could meet the publishing standards of the *PLOS one*.

Thank you very much for your assistance with our paper.

**Responses to Reviewer #2:**

*1.* *The logic of the literature review is not clear enough. The author should pay attention to the following questions: ① Is high-quality development equivalent to sustainable development? If there are differences between the two, the key words should be clearly defined in the process of literature review and discussion, and should not be arbitrarily replaced If the purpose of literature review is to clarify the factors that affect the high-quality development of resource-based cities, it should focus on sorting out the content rather than focusing on empirical methods.*

**Response:** According to the reviewer's comments, the following revisions are made to clarify the similarities and differences between high-quality development and sustainable development:

In addition, some scholars have conducted comparative studies on the relationship and differences between high-quality development and sustainable development, believing that early high-quality development was more focused on economic quality compared to sustainable development [30]. However, with the proposal of the five development concepts, the content and scope of high-quality development have continued to expand [31], leading to the gradual formation of a unified whole in which high-quality development and sustainable development are mutually coordinated and complementary.

*2.* *On the construction of the indicator system for high-quality development, the author has expanded the generalized high-quality indicator development system on the basis of the traditional high-quality development indicator system. However, the author has not clearly expressed the reasons and basis for the expansion of the article, and should be discussed in advance in the content of the literature review to make it reasonable to rely on.*

**Response:** According to the reviewer's comments, the following revisions are made to clarify the reasons and basis for the extension of high-quality development:

The academic community's understanding of the connotation of high-quality development has undergone a transformation from focusing solely on the quality of economic growth to development quality. Initially, people's understanding of high-quality development was mostly focused on economic development, and they believed that when measuring regional development, using only per capita GDP was not comprehensive enough, and that people's lives and social welfare should also be included [14, 15]. As scholars have conducted more in-depth research on the connotation of high-quality development, they have gradually incorporated aspects such as ecological environment, society, and human capital into the evaluation index system for measuring regional high-quality development [16-18]. At this point, the academic community has reached a consensus on the definition of the connotation of high-quality development, generally believing that high-quality development is efficient and high-quality development that can meet people's material, social, and psychological needs. Currently, with the proposal of the five development concepts of innovation, coordination, green, openness, and sharing, the connotation of high-quality development has been further deepened [19]. Compared with the previous connotation of high-quality development, the five development concepts have more systematic and comprehensive characteristics, more emphasis on people-oriented development, and are more in line with the trend of the times, providing more comprehensive theoretical support for evaluating regional development.

*3.* *For the selection of data years, the data from 2003 to 2018 are somewhat outdated. Although the author retains the integrity of the study area, it also greatly sacrifices the forward-looking nature of the article.*

**Response:** According to the reviewer's comments, the following explanation is provided for whether the data is outdated. The revised version is as follows:

First, the data from 2003 to 2018 can reflect the basic situation and development trend of China's resource-based cities. Therefore, these data are meaningful and do not lose their value because of their age. Second, because historical data can be used to study and predict future trends, these outdated data can also provide important clues for the article about possible future situations. Choosing data from these years does not necessarily sacrifice the forward-looking nature of the article. Finally, due to the outbreak of the COVID-19 epidemic in China in 2019, the data from 2019 to 2020 is relatively distorted, and some data from 2021 to 2022 has not yet been released, which is why the data from 2003 to 2018 is ultimately chosen.

*4.* *The author does not consider endogenous issues. Is there a reverse causal relationship between variables that affect high-quality development and high-quality development? Failure to consider endogenous issues is likely to cause significant errors in the coefficients of the article.*

**Response:** According to the reviewer's comments, an endogeneity test was conducted on whether there is a reverse causal relationship between the variables that affect high-quality development and high-quality development. The revised version is as follows:

4.2.3 Robustness test

To avoid the problem of reverse causality, following the approach of Zhu and Jing [62], this study selects the highest second-order and third-order spatial lag terms as instrumental variables and employs the generalized spatial two-stage least squares method to conduct an endogeneity test. The results of the robustness test regression are shown in Table 6, and the spatial lag term of high-quality development is significant at the 1% level. The regression results of each influencing factor are basically consistent with the baseline regression results, indicating that there is no reverse causality between the variables affecting high-quality development and high-quality development itself, and the baseline regression results are robust.

Table 6. Robustness test results

| **Variable** | **Second-order Lag** | **Third-order Lag** |
| --- | --- | --- |
| W*y | 0.082***（0.00） | 0.081***（0.00） |
| ln(IS) | 0.099（0.53） | 0.098（0.51） |
| ln(RD) | 0.073***（0.00） | 0.0731***（0.00） |
| ln(HR) | 0.088***（0.00） | 0.0876***（0.00） |
| ln(UPN) | 0.592***（0.00） | 0.592***（0.00） |
| ln(ER) | -0.006（0.107） | -0.006***（0.104） |
| ln(IEP) | 0.010***（0.00） | 0.010***（0.00） |
| ln(IT) | -0.013（0.75） | -0.013（0.74） |
| Observations | 1808 | 1808 |
| R-squared | 0.108 | 0.108 |
| Number of CODE | 113 | 113 |

Note: ***P < 0.01, **P < 0.05, *P < 0.1; the values in brackets are the corresponding z-statistics.
