# Supplementary material for: Spatiotemporal differentiation and analysis of factors influencing high-quality development of resource-based cities: An empirical study based in China
Source: PLoS One. 2024 May 31;19(5):e0304460. doi: 10.1371/journal.pone.0304460 (PMC11142578; doi:10.1371/journal.pone.0304460)
Supplement: Attachment — Submitted filename: PONE-D-23-05547_R1_Reviewer Comments.docx [file pone.0304460.s003.docx]

**REVIEWER CMMENTS**

**Manuscript Title:** Spatiotemporal differentiation and analysis of factors influencing high-quality development of resource-based cities: An empirical study based in China.

**Manuscript number:** PONE-D-23-05547_R1

Comments

The manuscript has been improved but it is recommended that authors work on the following suggestions:

1. The abstract is lengthy, I recommend that the author(s) focus on the salient points especially for the results.
2. In the introduction, line 75, kindly correct this: …, not only to solve their development problems but rather to …, not only to solve their developmental problems.
3. The objectives must be well stated.
4. The paragraph on the contribution of the study is not clear (line 95-106). Are the author(s) referring to the significance of the study?
5. The section on indicator system should be part of the literature while the specific indicators used and their ranges could be in the methods section.
6. Author(s) should check the version of ArcGIS used… it should be ArcGIS 10.2. This should be mentioned in the methods section and not the results. Stata is also mentioned in the results section but it would be more appropriate to mention the analytical software in the methods.
7. The version of ArcGIS used is not consistent. At a point, author(s) state it is 10.2 and in 10.3 at other points. Author(s) should kindly clarify this.
8. It is inappropriate to cite other scholarly works at the recommendations section. Author(s) should kindly revise this.
