# Supplementary material for: Spatiotemporal differentiation and analysis of factors influencing high-quality development of resource-based cities: An empirical study based in China
Source: PLoS One. 2024 May 31;19(5):e0304460. doi: 10.1371/journal.pone.0304460 (PMC11142578; doi:10.1371/journal.pone.0304460)
Supplement: Attachment — Submitted filename: Response to Reviewers.docx [file pone.0304460.s004.docx]

**Response to Reviewers**

**Manuscript Title:** Spatiotemporal differentiation and analysis of factors influencing high-quality development of resource-based cities: An empirical study based in China.

**Manuscript number:** PONE-D-23-05547_R1

**Comment 1:** *The abstract is lengthy; I recommend that the author(s) focus on the salient points especially for the results.*

**Response:** Thank you for the comment and suggestion. We have rechecked the abstract part of the paper and re-organized it carefully.

Line 24-39:

**Abstract：**Resource-based cities often face challenges such as lack of resources and insufficient power for transformation and complex high-quality development. Further, research on the factors influencing high-quality development in such cities is limited. Thus, our approach utilized the entropy weight TOPSIS method, spatial correlation analysis, and spatial econometric models to explore key factors influencing high-quality development in China's resource-based cities. Key findings include: (1) An annual increase in overall high-quality development among Chinese resource-based cities, with the growth rate ranking as mature > regeneration > growth > declining cities. (2) Positive spatial correlation in high-quality development across resource-based cities, with fluctuating Moran's I index results indicating varying levels of clustering. (3) Scientific and technological innovation, human capital, urbanization, and openness to the global market significantly impact high-quality development. Distinct variations in how these factors influence different types of resource-based cities. This empirical research offers valuable insights for policymakers working to reduce regional disparities and promote high-quality development in resource-based cities.

**Comment 2:** *In the introduction, line 75, kindly correct this: …, not only to solve their development problems but rather to …, not only to solve their developmental problems.*

**Response:** We agree with the reviewer’s comments and suggestions. We have carefully revised the part.

Line 63-65:

Therefore, promoting the high-quality development of resource-based cities is necessary, not only to solve their developmental problems but rather to implement a national strategy.

**Comment 3:** *The objectives must be well stated.*

**Response:** We agree with the reviewer’s comments and suggestions. We have carefully revised the objectives.

Line 74-86:

To address these gaps, our objective is to examine resource-based prefecture-level cities across China to systematically analyze the spatiotemporal evolutionary characteristics of high-quality development levels and the influencing mechanism for different types of resource-based cities. This paper takes 113 resource-based prefecture-level cities in China from 2003 to 2018 as samples, and uses entropy weight-TOPSIS method and spatial correlation analysis to construct a high-quality development evaluation system, and studies the spatio-temporal evolution trend of high-quality development in different types of cities. At the same time, a spatial econometric model is used to explore the role of industrial structure, technological innovation, human capital, urbanization, environmental regulation, openness to the outside world, and infrastructure in high-quality development. Finally, high-quality development suggestions are proposed based on empirical results.

**Comment 4:** *The paragraph on the contribution of the study is not clear (line 95-106). Are the author(s) referring to the significance of the study?*

**Response:** Thanks to the reviewer's comments. We have reorganized the contributions to this paper. The examples are as follows:

Line 87-100:

The contributions of this study are: (1) We have developed an inclusive and widely applicable evaluation index system for assessing high-quality development in resource-based cities. This innovative system, rooted in the "Five Major Development Concepts," fills a critical void in the current research landscape, providing a holistic framework for evaluation. (2) By encompassing the majority of China's resource-based cities within our research sample, our study offers a comprehensive analysis that holds practical relevance for a broader spectrum of regions. This inclusivity enhances the applicability of our findings. (3) Our research addresses the critical knowledge gap concerning the factors that influence high-quality development in resource-based cities. We meticulously select these influencing factors, employing a scientific and purposive approach. Through this comprehensive analysis, we shed light on the challenges these cities face during their high-quality development journey and offer well-informed solutions. This contribution advances our understanding of resource-based city development.

**Comment 5:** *The section on indicator system should be part of the literature while the specific indicators used and their ranges could be in the methods section.*

**Response：**We appreciate your valuable comments and recommended references on our manuscript. After revisions, we placed the indicator system in the literature review section and the specific indicators as well as the scope in the methods section.

Line 122-144:

Currently, with the proposal of the five development concepts of innovation, coordination, green, openness, and sharing, the connotation of high-quality development has been further deepened [19]. The five development concepts refer to the five development strategies proposed by the Communist Party of China in the context of the new era, namely innovation, coordination, green development, openness, and sharing. These concepts aim to guide the development of China's economy, politics, culture, and other fields, and promote China's transition to high-quality development [38]. Specifically, innovation refers to promoting technological and industrial innovation to improve the quality and efficiency of economic development; coordination refers to promoting coordinated development between urban and rural areas, industries, regions, and sectors; green development refers to promoting ecological civilization construction, protecting the environment, and achieving coordinated development between economic development and environmental protection; openness refers to deepening opening-up, actively participating in global economic governance, and promoting economic globalization; sharing refers to promoting social fairness and justice, strengthening social security, and achieving more equitable and inclusive distribution of development outcomes. The Five Development Concepts are interconnected and mutually reinforcing, forming an integrated whole with inherent connections [20]. Compared with the previous connotation of high-quality development, the five development concepts have more systematic and comprehensive characteristics, more emphasis on people-oriented development, and are more in line with the trend of the times, providing more comprehensive theoretical support for evaluating regional development.

Table 1. Evaluation index system and weight of high-quality development of resource-based cities

| **Criterion layer** | | | **Serial number** | **Indicator layer** | **Entropy Weight Method Weight** | **Indicator properties** |
| --- | --- | --- | --- | --- | --- | --- |
| **High-quality development based on "five development concepts"** | economy | growth efficiency | X_1_ | GDP/social workers | 0.025 | + |
|  |  |  | X_2_ | GDP growth rate | 0.037 | + |
|  |  |  | X_3_ | Growth rate of social fixed asset investment | 0.03 | + |
|  |  | growth level | X_4_ | Real GDP per capita | 0.029 | + |
|  |  |  | X_5_ | per capita fiscal revenue | 0.034 | + |
|  |  |  | X_6_ | gross industrial output per capita | 0.028 | + |
|  | innovation | innovative research and development | X_7_ | Science and technology expenditure | 0.032 | + |
|  |  |  | X_8_ | Number of granted patent applications | 0.023 | + |
|  |  |  | X_9_ | The number of green invention patents | 0.029 | + |
|  |  | innovation potential | X_10_ | Education spending as a share of GDP | 0.03 | + |
|  |  |  | X_11_ | Number of teachers per 10,000 people | 0.019 | + |
|  |  | transformation development | X_12_ | Energy consumption per ten thousand yuan of GDP | 0.025 | - |
|  |  |  | X_13_ | Employees in the tertiary industry among 10,000 people | 0.045 | + |
|  | coordination | regional coordination | X_14_ | Thiel Index | 0.031 | + |
|  |  | demand coordination | X_15_ | Total per capita consumption | 0.031 | + |
|  |  |  | X_16_ | Engel's coefficient of urban residents | 0.024 | - |
|  |  |  | X_17_ | Year-end urban registered unemployment rate | 0.032 | - |
|  |  | industrial structure | X_18_ | Primary and secondary industry coordination degree | 0.027 | - |
|  |  |  | X_19_ | Primary and tertiary industry coordination degree | 0.027 | - |
|  |  |  | X_20_ | Industrial development potential | 0.025 | + |
|  | green | resource consumption | X_21_ | Energy consumption per capita | 0.021 | - |
|  |  |  | X_22_ | Household water consumption per capita | 0.017 | + |
|  |  | ecological environment | X_23_ | Total industrial "three wastes" emissions per 10,000 yuan of industrial added value / t 10,000 yuan -1 | 0.017 | - |
|  |  |  | X_24_ | PM2.5 | 0.02 | - |
|  |  |  | X_25_ | Green coverage rate of built-up area | 0.025 | + |
|  |  | environmental governance | X_26_ | Comprehensive utilization rate of industrial solid waste | 0.017 | + |
|  |  |  | X_27_ | Centralized treatment rate of sewage treatment plant | 0.022 | + |
|  |  | green living | X_28_ | Green area per capita | 0.028 | + |
|  |  |  | X_29_ | Harmless treatment rate of domestic pollution | 0.023 | + |
|  | open | trade open | X_30_ | Total import and export trade per capita | 0.02 | + |
|  |  | investment open | X_31_ | Utilize the total amount of foreign investment/GDP | 0.034 | + |
|  |  |  | X_32_ | Number of foreign-invested enterprises | 0.027 | + |
|  | shared | public service | X_33_ | Per capita education expenditure of local finance | 0.031 | + |

**Comment 6:** *Author(s) should check the version of ArcGIS used… it should be ArcGIS 10.2. This should be mentioned in the methods section and not the results. Stata is also mentioned in the results section but it would be more appropriate to mention the analytical software in the methods.*

**Response：**We appreciate the reviewer’s helpful and detailed comments. We check the version of ArcGIS and determine it to be ArcGIS 10.2, and make corresponding changes in the text. The use of Stata is mentioned in the methods section.

Line 373-375:

The spatial pattern changes of high-quality development level of resource-based cities were analyzed using ArcGIS 10.2 software to examine the trend of hierarchical spatial distribution (Fig. 2).

Line 432-433:

This study used ArcGIS10.2 and Geoda1.14 to calculate the adjacency weight matrix.

Line 336-337:

The data processing software used in this paper is Stata15.0.

Line 449-452:

Stata15.0 software was used to draw Moran's I scatterplot of the high-quality development level of 113 resource-based prefecture-level cities in China, and Geoda1.14 was used to draw the LISA cluster map in local Moran's I to analyze its spatial agglomeration characteristics.

Line 483:

This study used Stata15.0 to select the analysis model (Table 4).

**Comment 7:** *The version of ArcGIS used is not consistent. At a point, author(s) state it is 10.2 and in 10.3 at other points. Author(s) should kindly clarify this.*

**Response：**Thanks to the reviewer's comments, we used ArcGIS 10.2 software and we rechecked the article and revised it carefully.

Line 373-375:

The spatial pattern changes of high-quality development level of resource-based cities were analyzed using ArcGIS 10.2 software to examine the trend of hierarchical spatial distribution (Fig. 2).

Line 432-433:

This study used ArcGIS10.2 and Geoda1.14 to calculate the adjacency weight matrix.

**Comment 8:** *It is inappropriate to cite other scholarly works at the recommendations section. Author(s) should kindly revise this.*

**Response：**Thank you for the excellent suggestions. We have restructured the recommendations section following your suggestions. The revised Conclusions and recommendations section are provided below for your convenience.

Line 676-693:

Based on our results, we propose the following policy recommendations: (1) Drive innovation and upgrade industries. Strengthen innovation as a driver for upgrading traditional resource-based industries with green technology. Enhance the technology innovation system, focus on human capital investment, and cultivate independent innovation. (2) Promote interregional resource flow. Improve interregional coordination for efficient resource circulation between urban and rural areas, fostering conditions for industrial upgrades. (3) Enhance pollution control and green tech. Strengthen coordinated pollution control by improving energy efficiency and environmental practices, minimizing spillover effects between cities, and encouraging the use of green technologies. (4) Boost openness and investment. Elevate openness levels through strategic investment attraction in resource-saving and environmentally friendly enterprises, promoting energy-saving technologies and cross-border cooperation. (5) Establish shared development system. Build a shared development system through new infrastructure like "5G" to enhance public services, education, healthcare, and infrastructure investment efficiency. (6) Optimize human capital and transformation. Enhance human capital in resource-based cities through higher education, supporting transformation towards resource-based and innovative industries, and optimizing talent resource allocation.
