# Supplementary material for: Spatiotemporal differentiation and analysis of factors influencing high-quality development of resource-based cities: An empirical study based in China
Source: PLoS One. 2024 May 31;19(5):e0304460. doi: 10.1371/journal.pone.0304460 (PMC11142578; doi:10.1371/journal.pone.0304460)
Supplement: Attachment — Submitted filename: Response to Reviewers.docx [file pone.0304460.s005.docx]

**Response to the Review Team**

**PONE-D-23-05547R2**

**Spatiotemporal differentiation and analysis of factors influencing high-quality development of resource-based cities: An empirical study based in China**

Dear editor and reviewers,

We greatly appreciate the opportunity to revise our paper, as well as the time and effort that the Academic Editor, Professor Jing Cheng, and the two reviewers have invested in providing constructive and valuable feedback. Following the review team’s suggestions, we have carefully revised our paper as detailed in our point-to-point response below. We are grateful for the feedback and believe that the paper has significantly improved both in content and organization. We hope that you are satisfied with our effort and that you will find the revised manuscript acceptable for publication in the *PLOS ONE.*

Thank you very much for your assistance with our paper.

The sponsor of this article is Liu Xiuli, and the role of the article is project administration and funding acquisition.

For ease of your review, the main changes in the text content of the paper are tracked in **BLUE**.

**Response to Reviewer 1**

**General comment:** *This paper utilized the entropy weight TOPSIS method, spatial correlation analysis, and spatial econometric models to explore key factors influencing high-quality development in China's resource-based cities. The author executed a full revision and I recommend its acceptance.*

**Response:** We are pleased to know that you recommend the acceptance of the revised manuscript. Your feedback has been invaluable in enhancing the quality of our research. Thank you for your time and constructive feedback.

**Response to Reviewer 3**

**Comment 1**: *The bulleting of the results should be removed.*

**Response:** Thank you very much for your meticulous review to improve the format of our paper. Based on your suggestion, we have removed the bulleting of the results.

**Comment 2**: Some paragraphs are too long, especially at the introduction section and must be reduced.

**Response:** Thank you very much for your thoughtful and constructive suggestions for improving the paper. Based on your suggestions, we made it more readable by splitting some paragraphs in the article and modifying the sentences in the paragraphs, especially the introduction.

**1. Introduction**

China's economy has shifted from high-speed growth to a stage of high-quality development[1]. This high-quality development is a crucial requirement for current and future regional development[2]. However, approximately 40% of China's cities are resource-based, serving as vital energy security bases that significantly contribute to the country's economic and social progress. However, owing to the non-renewable nature of resources, high-quality development of several resource-based cities can be challenging. They face problems resulting from single industrial structure, slow economic growth, insufficient innovation ability, a lack of energy resources, deterioration of the ecological environment, and increasing pressure on people's livelihoods[3-4].

To address these challenges, China issued several opinions in 2007 to promote the sustainable development of resource-based cities, emphasizing the establishment and improvement of a long-term mechanism for development[5]. In 2013, the national sustainable development plan for resource-based cities (2013–2020)[6] proposed sustainable development paths for these cities. In 2017, further emphasis was placed on the transformation and development of resource-based cities, highlighting high-quality development aligned with the five development concepts of "innovation, coordination, green, openness, and sharing." The 19th CPC National Congress report pointed out that sufficient support should be provided for the transformation and development of resource-based cities. Therefore, promoting the high-quality development of resource-based cities is necessary, not only to solve their developmental problems but also to implement a national strategy.

Currently, research in academia on high-quality development mainly focuses on its connotation, meaning, and evaluation [7-9], and a unified evaluation index system for high-quality development has not yet been formed, especially for resource-based regions. There is a lack of research on the evaluation index system for high-quality development in resource-based regions. In addition, scholars often study resource-based cities at national, provincial, and urban levels, examining aspects like labor quality[10], environmental pollution[11-12], and education quality[13], but research on factors influencing high-quality development remains limited.

To address these gaps, our objective is to examine resource-based prefecture-level cities across China to systematically analyze the spatiotemporal evolutionary characteristics of high-quality development levels and the influencing mechanism for different types of resource-based cities. This paper takes 113 resource-based prefecture-level cities in China from 2003 to 2018 as samples, uses the entropy weight-TOPSIS method and spatial correlation analysis to construct a high-quality development evaluation system, and studies the spatio-temporal evolution trend of high-quality development in different types of cities. At the same time, a spatial econometric model is used to explore the role of industrial structure, technological innovation, human capital, urbanization, environmental regulation, openness to the outside world, and infrastructure in high-quality development. Finally, high-quality development suggestions are proposed based on empirical results.

The contribution of this study is mainly reflected in three aspects: firstly, this study develops an inclusive and widely applicable evaluation index system for evaluating the high-quality development of resource-based cities. This innovative system is based on the "Five Development Concepts", fills important gaps in current research fields, and provides a comprehensive framework for assessment. Secondly, by including most of China's resource-based cities in the study sample, this study provides a comprehensive analysis with practical relevance covering a wider region. This helps improve the applicability of the research results. Lastly, the study meticulously selects relevant influencing factors through a scientific and purposive approach, thereby filling crucial knowledge gaps concerning the factors influencing high-quality development in resource-based cities. Through this comprehensive analysis, the study reveals the challenges that resource-based cities face during their high-quality development journey and provides informed solutions, contributing to a better understanding of the essence of high-quality development in resource-based cities for policymakers.

The rest of this study is structured as follows. Part 2 introduces the relevant literature on high-quality development in resource-based cities. Part 3 describes the research methods, study areas, data sources, and variable selection. Part 4 presents the research structure and analysis, including spatio-temporal evolution analysis of high-quality development in resource-based cities and empirical testing of influencing factors. Part 5 is the conclusion and policy implications of the study.

**Comment 3**: *The contribution of the study should not be numbered.*

**Response:** Thank you for the comment and suggestion. We have restructured the study contributions to be unnumbered as follows:

***Line 89:*** The contribution of this study is mainly reflected in three aspects: firstly, this study develops an inclusive and widely applicable evaluation index system for evaluating the high-quality development of resource-based cities. This innovative system is based on the "Five Development Concepts", fills important gaps in current research fields, and provides a comprehensive framework for assessment. Secondly, by including most of China's resource-based cities in the study sample, this study provides a comprehensive analysis with practical relevance covering a wider region. This helps improve the applicability of the research results. Lastly, the study meticulously selects relevant influencing factors through a scientific and purposive approach, thereby filling crucial knowledge gaps concerning the factors influencing high-quality development in resource-based cities. Through this comprehensive analysis, the study reveals the challenges that resource-based cities face during their high-quality development journey and provides informed solutions, contributing to a better understanding of the essence of high-quality development in resource-based cities for policymakers.

**Comment 4**: *Table 1 should be well formatted.*

**Response:** Thank you very much for your thoughtful and constructive suggestions to improve this article. Based on your suggestions, we have optimized and adjusted the format of Table 1 by deleting the "Serial number" column and adjusting the font size in the table, as shown below.

**Line 188:**

Table 1. Evaluation index system and weight of high-quality development of resource-based cities

| Criterion layer | | | Indicator layer | Entropy Weight Method Weight | Indicator properties |
| --- | --- | --- | --- | --- | --- |
| High-quality development based on "five development concepts" | economy | growth efficiency | GDP/social workers | 0.025 | + |
|  |  |  | GDP growth rate | 0.037 | + |
|  |  |  | Growth rate of social fixed asset investment | 0.03 | + |
|  |  | growth level | Real GDP per capita | 0.029 | + |
|  |  |  | per capita fiscal revenue | 0.034 | + |
|  |  |  | gross industrial output per capita | 0.028 | + |
|  | innovation | innovative research and development | Science and technology expenditure | 0.032 | + |
|  |  |  | Number of granted patent applications | 0.023 | + |
|  |  |  | The number of green invention patents | 0.029 | + |
|  |  | innovation potential | Education spending as a share of GDP | 0.03 | + |
|  |  |  | Number of teachers per 10,000 people | 0.019 | + |
|  |  | transformation development | Energy consumption per ten thousand yuan of GDP | 0.025 | - |
|  |  |  | Employees in the tertiary industry among 10,000 people | 0.045 | + |
|  | coordination | regional coordination | Thiel Index | 0.031 | + |
|  |  | demand coordination | Total per capita consumption | 0.031 | + |
|  |  |  | Engel's coefficient of urban residents | 0.024 | - |
|  |  |  | Year-end urban registered unemployment rate | 0.032 | - |
|  |  | industrial structure | Primary and secondary industry coordination degree | 0.027 | - |
|  |  |  | Primary and tertiary industry coordination degree | 0.027 | - |
|  |  |  | Industrial development potential | 0.025 | + |
|  | green | resource consumption | Energy consumption per capita | 0.021 | - |
|  |  |  | Household water consumption per capita | 0.017 | + |
|  |  | ecological environment | Total industrial "three wastes" emissions per 10,000 yuan of industrial added value / t 10,000 yuan -1 | 0.017 | - |
|  |  |  | PM2.5 | 0.02 | - |
|  |  |  | Green coverage rate of built-up area | 0.025 | + |
|  |  | environmental governance | Comprehensive utilization rate of industrial solid waste | 0.017 | + |
|  |  |  | Centralized treatment rate of sewage treatment plant | 0.022 | + |
|  |  | green living | Green area per capita | 0.028 | + |
|  |  |  | Harmless treatment rate of domestic pollution | 0.023 | + |
|  | open | trade open | Total import and export trade per capita | 0.02 | + |
|  |  | investment open | Utilize the total amount of foreign investment/GDP | 0.034 | + |
|  |  |  | Number of foreign-invested enterprises | 0.027 | + |
|  | shared | public service | Per capita education expenditure of local finance | 0.031 | + |

**Comment 5**: *Remove ‘change’ from line 371.*

**Response:** Thank you very much for your detailed review and for motivating us to push our work further. We have deleted the change in line 371, as shown below:

***Line 359:*** *The spatial patterns of high-quality development level of resource-based cities are analyzed using ArcGIS 10.2 software to examine the trend of hierarchical spatial distribution.*

**Comment 6**: *Results paragraph should not be numbered.*

Response: Thank you very much for your thoughtful and constructive suggestions to improve this article. Based on your suggestions, we have restructured the results paragraphs to be unnumbered and reformatted, please see the manuscript for details.

**Comment 7**: *The manuscript did not have a discussions section (relating findings with existing literature) after the results.*

**Response:** Thank you for organizing an excellent review, for your support, and for providing us with clear revision guidelines. Based on your suggestions, we have added a discussion in the fifth part. The writing ideas in the discussion part are mainly based on the research purpose of the article. By analyzing the similarities and differences between the research conclusions of the article and the existing literature, we can draw further research directions in the future, as follows:

***Line 642:***

**5.2 Discussion**

Based on the five major development concepts, we examine the high-quality development of resource-based cities across China by constructing a complete high-quality development evaluation index system, and systematically analyze the spatio-temporal evolution characteristics of high-quality development levels and the impact mechanisms of different types of resource-based cities.

The study reveals that resource-based cities exhibit an upward trend in the level of high-quality development. Significant variations in high-quality development are observed among different regions, consistent with findings by Su[54] and Cui et al.[55]. Furthermore, spatial correlation analysis indicates an overall positive spatial correlation in the high-quality development of resource-based cities, confirming results from Ding and Liu[56]. Notably, there is a distinct spatial spillover effect in high-quality development. Exploring the influencing mechanisms of high-quality development in resource-based cities, it is found that levels of technological innovation, human capital, urbanization, and openness significantly promote high-quality development[57]. Conversely, environmental regulation exerts a notable inhibitory effect, contrary to the results of Li et al.[58],. This discrepancy may be attributed to the threshold effect of environmental regulation, leading to variations in conclusions at different study periods.

The differences between this study and the existing literature also point out the direction for future research. In the future, further exploration will be conducted in two aspects: first, the reasons for the differences in the impact mechanisms studied in this paper and those of other scholars should be analyzed, and it may be related to the existence of threshold effects. Therefore, future research should focus on whether there are threshold effects in the impact mechanism. In addition, facing various factors such as the proposal of China's "dual carbon" goal and the construction of the "14th Five-Year Plan", there is still a large space for promoting and improving the high-quality development of resource-based cities. The next step of the research should take these factors into consideration and solve current and future practical problems.

**Thank you once again for your careful reading of the paper, for your support, and your constructive comments. We enjoyed working on your suggestions! We hope that you are satisfied with our efforts and find the revised version acceptable.**
