# Supplementary material for: Spatiotemporal differentiation and analysis of factors influencing high-quality development of resource-based cities: An empirical study based in China
Source: PLoS One. 2024 May 31;19(5):e0304460. doi: 10.1371/journal.pone.0304460 (PMC11142578; doi:10.1371/journal.pone.0304460)
Supplement: Attachment — Submitted filename: Response to Reviewers.docx [file pone.0304460.s006.docx]

**Response to the Review Team**

**PONE-D-23-05547_R1**

**Spatiotemporal differentiation and analysis of factors influencing high-quality development of resource-based cities: An empirical study based in China**

Dear Editors and Reviewers,

We greatly appreciate the opportunity to revise our paper, as well as the time and effort that the Editor-in-Chief and the reviewer have invested in providing constructive and valuable feedback. Following the review team’s suggestions, we have carefully revised our paper as detailed in our point-to-point response below. We are grateful for the feedback and believe that the paper has significantly improved both in content and organization. We hope that you are satisfied with our effort and that you will find the revised manuscript acceptable for publication in the *PLOS ONE*.

For ease of your review, the main changes in the text content of the paper are tracked in **BLUE**.

**Response to Reviewer 1**

**General comment:** *The manuscript has been improved but it is recommended that authors work on the following suggestions.*

**Response:** We greatly appreciate the reviewer’s assessment of our work and the helpful comments. The manuscript has been carefully revised according to the reviewer’s comments.

**Comment 1:** *The abstract is lengthy, I recommend that the author(s) focus on the salient points especially for the results.*

**Response:** Thank you for the comment and suggestion. We have rewritten the abstract section to highlight the results section of the paper.

Line 24-40:

**Abstract**

Resource-based cities often face problems such as resource scarcity and insufficient electricity to achieve complex high-quality growth. At present, there is relatively little research on the impact on the high-quality development of such cities. To study the key variables that affect the high-quality growth of resource-based cities, we adopt entropy weighted TOPSIS technology, spatial correlation analysis, and spatial econometric models. The main conclusions are as follows: (1) The overall high-quality development of resource-based cities in China is on the rise year by year; The cities with the highest growth rates are those that are mature, rejuvenated, growing, and declining. (2) Resource-based cities have a positive geographical correlation in high-quality development, and different numbers of clusters are displayed by changing the Moran I index score. (3) High quality development is strongly influenced by human capital, urbanization, technological innovation, and global market openness. There are significant differences in the ways in which these variables affect various types of resource-based cities. Policy makers who strive to reduce regional inequality and encourage high-quality growth in resource-based communities may benefit greatly from the insights provided by this study.

**Comment 2:** *In the introduction, line 75, kindly correct this: …, not only to solve their development problems but rather to …, not only to solve their developmental problems.*

**Response:** Thank you for your careful review, we have revised it for your review.

Line 65-69:

The 19th CPC National Congress report pointed out that sufficient support should be provided for the transformation and development of resource-based cities. Therefore, promoting the high-quality development of resource-based cities is necessary, not only to solve their developmental problems but also to implement a national strategy.

**Comment 3:** *The objectives must be well stated.*

**Response:** We thank the reviewer for bringing this to our attention. We have rewritten this paper to describe the objectives of this paper more accurately.

Line78-88:

To fill these gaps, our goal is to examine resource-based cities across China, systematically analyze the spatiotemporal evolution characteristics of high-quality development levels, and the impact mechanisms of different types of resource-based cities. This article takes 113 resource-based cities in China from 2003 to 2018 as samples, uses entropy weighted TOPSIS method and spatial correlation analysis to construct a high-quality development evaluation system, and studies the spatiotemporal evolution trends of high-quality development in different types of cities. Meanwhile, using spatial econometric models, this study explores the roles of industrial structure, technological innovation, human capital, urbanization, environmental regulation, openness, and infrastructure in high-quality development. Finally, based on empirical results, high-quality development suggestions were proposed.

**Comment 4:** *The paragraph on the contribution of the study is not clear (line 95-106). Are the author(s) referring to the significance of the study?*

**Response:** Thanks for kindly reminding us to clarify this point. The contribution of this paragraph refers to the significance of this study. We have rewritten the contribution of this study to make it clearer and more focused.

Line 89-102:

The contribution of this study is mainly reflected in three aspects: firstly, this study developed an inclusive and widely applicable evaluation index system for the high-quality development of resource-based cities. This innovative system is based on the “Five Development Concepts” and fills an important gap in the current research field, providing a comprehensive framework for evaluation. Secondly, by incorporating most resource-based cities in China into the research sample, this study provides a comprehensive analysis with practical significance covering a wider range of regions. This helps to improve the applicability of research results. Finally, this study selected relevant influencing factors through scientific and targeted methods, thus filling the research gap in the factors affecting the high-quality development of resource-based cities. Through this comprehensive analysis, this study reveals the challenges faced by resource-based cities in the process of high-quality development and provides wise solutions, which helps decision-makers better understand the essence of high-quality development in resource-based cities.

**Comment 5:** *The section on indicator system should be part of the literature while the specific indicators used and their ranges could be in the methods section.*

**Response:** We agree with the reviewer’s point. Based on your suggestions, we present the indicator system in the literature review section and the specific indicators in the methodology section.

Line 179-187:

**3.1.1 Measurement of high-quality development level of resource-based cities**

**(1) Indicator system.** This study is based on “five development concepts”. It also draws on relevant research on sustainable development[39], human development evaluation[40], high-quality development in China in the new era[41-43], and resource-based city transformation[44]. According to the special objective situation of resource-based cities[44], this study establishes a high-quality development index system for resource-based cities. Six subsystem evaluations of economy, innovation, coordination, greenness, openness, and sharing are also constructed. Using the entropy weight method, the weight of each evaluation index is determined (Table 1).

**Comment 6:** *Author(s) should check the version of ArcGIS used… it should be ArcGIS 10.2. This should be mentioned in the methods section and not the results. Stata is also mentioned in the results section but it would be more appropriate to mention the analytical software in the methods.*

**Response:** Thank you for this excellent suggestion and for motivating us to push our work further. We have checked the version of ArcGIS as 10.2. and we have mentioned the software we are using in the methodology section.

Line 246-249:

**(2) Local spatial autocorrelation model.** As the global spatial autocorrelation Moran's I index cannot reflect the heterogeneity between regions, the local Moran's I index, namely the local index of spatial correlation (LISA), is introduced to reveal the relationship between resource-based and neighboring cities using ArcGIS 10.2 software.

Line 259-263:

**3.1.3 Spatial Econometric Model**

The high-quality development of resource-based cities is not only affected by these factors but also by the development of neighboring cities. Therefore, this study uses a spatial econometric model to conduct an empirical test of the factors affecting the high-quality development of resource-based cities using Stata 15.0 software.

Line 323-326:

Individual missing data are estimated using linear interpolation, and the data are logarithmically processed to eliminate the effect of heteroscedasticity. The data processing software used in this paper is Stata15.0. The descriptive statistics for the variables are presented in Table 2.

**Comment 7:** *The version of ArcGIS used is not consistent. At a point, author(s) state it is 10.2 and in 10.3 at other points. Author(s) should kindly clarify this.*

**Response:** We thank the reviewers for drawing our attention to this point. We have checked the version of the ArcGIS version for 10.2 and revised it throughout.

**Comment 8:** *It is inappropriate to cite other scholarly works at the recommendations section. Author(s) should kindly revise this.*

**Response:** Thanks to the reviewer for this careful comment. We have eliminated references to other scholars in the recommendations section.

**Line 671-689:**

**6. Policy implications**

Based on our results, we propose the following policy recommendations: (1) Drive innovation and upgrade industries. Strengthen innovation as a driver for upgrading traditional resource-based industries with green technology. Enhance the technology innovation system, focus on human capital investment, and cultivate independent innovation. (2) Promote interregional resource flow. Improve interregional coordination for efficient resource circulation between urban and rural areas, fostering conditions for industrial upgrades. (3) Enhance pollution control and green tech. Strengthen coordinated pollution control by improving energy efficiency and environmental practices, minimizing spillover effects between cities, and encouraging the use of green technologies. (4) Boost openness and investment. Elevate openness levels through strategic investment attraction in resource-saving and environmentally friendly enterprises, promoting energy-saving technologies and cross-border cooperation. (5) Establish a shared development system. Build a shared development system through new infrastructure like "5G" to enhance public services, education, healthcare, and infrastructure investment efficiency. (6) Optimize human capital and transformation. Enhance human capital in resource-based cities through higher education, supporting transformation towards resource-based and innovative industries, and optimizing talent resource allocation.

**Again, we highly appreciate the opportunity to revise and resubmit the manuscript again as well as your comments and suggestions on it. They have helped us improve the manuscript significantly. We hope we have answered all of your questions and have revised the manuscript to your satisfaction.**
